# A perturbation-based framework for link prediction via non-negative matrix factorization

**DOI:** 10.1038/srep38938

**Published:** 2016-12-15

**Authors:** Wenjun Wang, Fei Cai, Pengfei Jiao, Lin Pan

**Affiliations:** 1 School of Computer Science and Technology, Tianjin University, Tianjin, 300350, China; 2 School of Surveying and Geo-Informatics, Shandong Jianzhu University, Jinan, 250101, China; 3 School of Marine Science and Technology, Tianjin University, Tianjin, 300072, China

## Abstract

Many link prediction methods have been developed to infer unobserved links or predict latent links based on the observed network structure. However, due to network noises and irregular links in real network, the performances of existed methods are usually limited. Considering random noises and irregular links, we propose a perturbation-based framework based on Non-negative Matrix Factorization to predict missing links. We first automatically determine the suitable number of latent features, which is inner rank in NMF, by Colibri method. Then, we perturb training set of a network by perturbation sets many times and get a series of perturbed networks. Finally, the common basis matrix and coefficients matrix of these perturbed networks are obtained via NMF and form similarity matrix of the network for link prediction. Experimental results on fifteen real networks show that the proposed framework has competitive performances compared with state-of-the-art link prediction methods. Correlations between the performances of different methods and the statistics of networks show that those methods with good precisions have similar consistence.

Complex network has been a popular topic in the past decade and attracted the research interests of multiple disciplines, including computer science, social science, physical science and mathematical science^1^. Lots of real world systems can be represented as complex networks, where the entities become nodes and interacting entities are connected by edges. For example, in social networks, the nodes denote individuals and the edges represent the interaction or common interests; in collaboration networks, the nodes denote authors and the edges represent collaborative relationship^2^.

In general, link prediction estimates the probability of a link between two nodes based on the network structure^3^. Link prediction can not only help to analyze complex networks with missing links^4^, but also be used to predict the links which may appear in the future^5^. In biological networks, it is a fundamental problem to demonstrate whether there is a link between two nodes, which usually cost too much to do laboratorial experiments. Hence, it may largely reduce the experimental costs if we can infer the unobserved links based on the observed links with a certain prediction precision. In online social networks, link prediction can help to recommend friends or interests. Furthermore, link prediction has been applied into analyzing network evolution, detecting network anomalies, etc^6^,^7^.

There are two main classes of link prediction methods: similarity-based algorithms and probabilistic models^8^. By similarity-based algorithms, the unlinked node pair with higher similarity is supposed to be more likely to be linked. The similarity can be defined with a variety of indices, including local indices and global indices. For example, Common Neighbours (CN) index is defined as the number of common neighbours of the two nodes in the networks^9^, Jaccard index is defined as the number of common neighbours of two nodes divided by interaction set of their degrees^10^, Katz index is based on the ensemble of all paths between each node pair. Cannistraci-resource-allocation (CRA) is a powerful local and parameter-free similarity-based index for link prediction in both monopartite network and bipartite network, and it is based on the local-community-paradigm^11^,^12^, which is a theory recently proposed to model local-topology-dependent link-growth in complex networks. In brief, similarity-based indices can be local or global, parameter-free or parameter-dependent, simple or complex. However, the calculations of most similarity indices only use the information of the network topology. Probabilistic models or generated models are another series of powerful methods for link prediction. By constructing the generating model of complex networks, link prediction becomes a problem of parameter learning in the model, thus, the probability of the missing links can be predicted by the learned model^13^. Probabilistic Relationship Model (PRM) defines a joint probability distribution over all the features of the networks^14^. Hierarchical structure model assumes that real networks are hierarchical and can be divided into different groups with subgroups^15^. Stochastic block model (SBM) assumes the relations between nodes are only dependent on the groups the nodes belong to. SBM has also been used to study the community detection and role identification of complex networks^16^. Probabilistic models have many advantages in network analysis and real applications. However, parameters learning and inference is a tricky problem.

Matrix factorization approach is a method that is to learn latent features from the network data for link prediction^17^,^18^,^19^. In a network, the nodes can be projected in a latent space and the probability of the edges depends on the nodes’ positions in this space. Each feature of the latent space is regarded as a latent attribute^20^, and two nodes are more likely similarity if they have similar latent features^21^. From another point of view, the similarity matrix of a complex network can be approximated to the product of two matrixes with lower features, which are basis matrix and coefficients matrix respectively. If we restrict the elements of the two matrixes to be non negative, the solution can be obtained by the algorithm of Non-negative Matrix Factorization^22^. However, it is difficult to automatically determine the number of latent features.

Real networks are made up of predictable regularity and unpredictable components. In the view of this situation, Structural Perturbation Method (SPM) that predicts the missing links by perturbed eigenvectors was proposed^23^. SPM method is based on the hypothesis that eigenvector is invariant and eigenvalues have the tiny perturbation when perturbation occurs in network. SPM reconstructs perturbed network by the small change of eigenvalues. However, it doesn’t consider the intrinsic nature that unpredictable components are made up of random noises and irregular links.

The existence of unpredictability components makes the best prediction accuracy unlikely to be 1 in real network. For instance, in the formation of real social network, friends usually know each other via their friends. The more friends they share, the more possibility that they will become friends. This way is formed through mechanistic models, such as CN, Salton and Jaccard index. However, there exist network noises in social network, that is to say, a small portion of the network we have observed is illusions made by network noises. Apart from noises, there are also unpredictable but real links. For example, two people, sharing no common friends, become friends in an accidental emergency, which cannot be explained by some generative models in link prediction. Due to the network noises and irregular links in real network, the prediction accuracy is usually limited. In this paper, a perturbation framework based on non-negative matrix factorization is proposed. The procedures of our framework are as follows. Firstly, the observed network is randomly divided into two separated parts, which are known as a training set and a test set respectively. Secondly, the suitable number of latent features *K* is automatically determined by Colibri method^24^. If *K* is overlarge, latent space model will be overfit of training set; if *K* is too small, the model will be underfit of training set. Therefore, it is necessary to automatically determine a suitable *K* value, meanwhile, Colibri method provides us with a very good choice because of its high efficiency and easy to extend to large scale networks. Thirdly, the training set is perturbed by small perturbation sets many times, and we get a series of perturbed networks. The perturbation mechanism of random deleting links is adopted aiming at tackling the problem of random noise in the network; the perturbation mechanism of random adding links is employed with the intention to handle the real but irregular links. Fourthly, the common basis matrix and coefficients matrix are learned from the perturbed networks via non-negative matrix factorization (NMF). In NMF, two popular distance, namely Euclidean distance (the square of the Frobenius norm) and Kullback-Leibler divergence, are adopted to construct objective function in the framework. Finally, based on the common basis matrix and coefficients matrix, we can obtain the similarity matrix, which is used to evaluate the result of link prediction. The experiments on eleven real-world network validate the effectiveness of this framework.

## Results

In this section, we first introduce the basic principle of perturbation-based framework by NMF (see Methods section for details). Next we introduce the evaluation metrics and baseline methods to be compared. Then we give experimental results on eleven real networks and in-depth analysis.

Consider undirected and unweighted network *G* = (*V, E*), where *V* and *E* are the set of nodes and the set of links, respectively. The number of nodes is denoted as *N* and the number of links is denoted as *M*. The given network can be represented by *A* ∈ {0, 1}^*N*×*N*^, where the element *A*_*ij*_ = 1 if nodes *i* and *j* are connected; otherwise, *A*_*ij*_ = 0.

### The basic principle of perturbation-based framework by NMF

**Figure 1:**
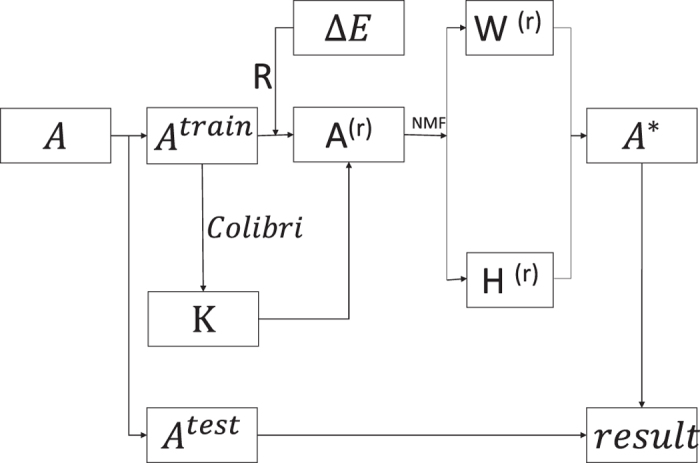
Perturbation-based framework by NMF. *A*^*train*^ is adjacency matrix of training set, *A*^*test*^ is adjacency matrix of test set, *K* is the number of latent features, Δ*E* is perturbation set, *A*^(*r*)^ is new perturbed matrix, *W*^(*r*)^ is basis matrix, *H*^(*r*)^ is coefficients matrix and A* is similarity matrix of original network.

We propose a perturbation-based framework by NMF, which is shown in Fig. 1. For a given network, we randomly divide the observed link set *E* into a training set *E*^*train*^ and a test set *E*^*test*^. The number of links of *E*^*train*^ is *M* − *L* and the number of links of *E*^*test*^ is *L. A*^*train*^ ∈ {0, 1}^*N*×*N*^ and *A*^*test*^ ∈ {0, 1}^*N*×*N*^ represent the adjacency matrix of the training set and the adjacency matrix of the test set, respectively. The number of the latent features *K* is automatically optimized by Colibri method in *A*^*train*^. Then we construct a perturbation set Δ*E* to perturb *E*^*train*^ by *R* times and get a series of new perturbed matrixes 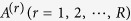. Based on the new perturbed matrices 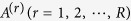 and *K*, we obtain the basis matrix *W*^(*r*)^ and the coefficients matrix *H*^(*r*)^. Finally, we get the similarity matrix of the original network with 
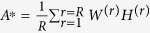
.

There are two ways to construct perturbation sets Δ*E* and the corresponding adjacent matrix *A*^Δ^, one is called random deletion perturbation, the other is called random addition perturbation. Random deletion perturbation is adopted aiming at tackling the problem of random noises in the network, while random addition perturbation is employed with the intention to handle the real but irregular links. Construction process of Δ*E* and the corresponding adjacent matrix *A*^Δ^ by random deletion perturbation is as follows:

- Step 1: Define a parameter *η* as the perturbation ratio on *E*^*train*^;
- Step 2: Randomly select *η*(*M* − *L*) links, which will be removed from *E*^*train*^ in the perturbation step, to construct Δ*E*;
- Step 3: Perturb *E*^*train*^ by Δ*E*, obviously, *A*^(*r*)^ = *A*^*train*^ − *A*^Δ^;
- Step 4: Independently repeat step 2 and step 3 for *R* times and obtain 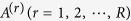.
- The construction process of Δ*E* and the corresponding adjacent matrix *A*^Δ^ by random addition perturbation is as follows:
- Step 1: Define a parameter *η* as the perturbation ratio on *E*^*train*^;
- Step 2: Denote the universal set of links as *U*. Randomly select *η*(*M* − *L*) links from *U* − *E*^*train*^, which will be added to *E*^*train*^ in the perturbation step, as Δ*E*. Obviously, *A*^(*r*)^ = *A*^*train*^ − *A*^Δ^;
- Step 3: Perturb *E*^*train*^ by Δ*E*, obviously, *A*^(*r*)^ = *A*^*train*^ + *A*^Δ^;
- Step 4: Independently repeat step 2 and step 3 for *R* times and obtain 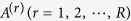.

Similarly to NMF, we propose two different cost functions. The first cost function with the square of the Euclidean distance can be written as


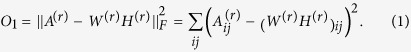


The second cost function with Kullback-Leibler divergence can be written as


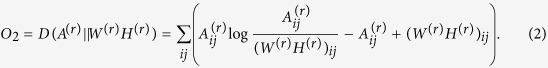


By minimizing the two cost functions *O*_1_ and *O*_2_, we get the basis matrix *W*^(*r*)^ and the coefficients matrix *H*^(*r*)^. At last, we get the similarity matrix of the original network with 
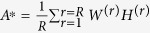
. Details can be seen in Method section.

Based on our framework, we propose four methods which are called *NMF* − *D*1, *NMF* − *A*1, *NMF* − *D*2 and *NMF* − *A*2, respectively. Here, *NMF* − *D*1 denotes method which optimize cost function *O*_1_ with random deletion perturbation. *NMF* − *A*1 denotes method which optimize cost function *O*_1_ with random addition perturbation. *NMF* − *D*2 denotes method which optimize cost function *O*_2_ with random deletion perturbation. *NMF* − *A*2 denotes method which optimize cost function *O*_2_ with addition deletion perturbation.

Our proposed methods, including *NMF* − *D*1, *NMF* − *A*1, *NMF* − *D*2 and *NMF* − *A*2, are not parameter-free. In addition to selection of the cost functions to optimize, there are two parameters that are perturbation ratio *η* and perturbation times *R* that should be tuned. Here, default value of perturbation ratio *η* is 0.1, and the default value of perturbation times *R* is 20. This is because that the probability value of a unperturbed link, which is (1 − 0.1)^20^ ≈ 0.1215, is very small. So default values of *η* and *R* can ensure that every link can randomly be selected into perturbation set Δ*E*.

### Evaluation Metrics

Precision and relative precision are considered in this paper. AUC (area under the receiver operating characteristic curve) and precision are the two widely used evaluation metrics for link prediction^8^. However, recent works^25^,^26^ clearly demonstrate that AUC is a deceptive measure for the evaluation of link prediction. The reasons are as follows: firstly, AUC needs the definition of a negative set, which is composed by all the missing (unobserved) links in the network except for the removed links (for test) that compose the positive set. However, in reality a negative set in the link prediction problem does not exist, and the link prediction is not a classification problem, thus it cannot be evaluated using AUC. Secondly, if AUC is a classification problem, the number of negative set would be 
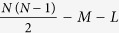
 and the number of positive set would be *L*, where *L* would be the number of test set. In sparse networks, the number of negative set would be much larger than the number of positive set. It is biased towards a negative set that is predominant on the positive set (removed links). Furthermore, AUC will give more importance to methods that overfit the network structure rather than offer a more general prediction ability. On the contrary, precision represent a better solution for link prediction. Given the ranking of the unobserved links, precision is defined as


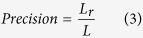


where *L* is the number of the predicted links, i.e. the number of links in *A*^*test*^, *L*_*r*_ is the number of correctly predicted links. Thus, higher precision means higher prediction accuracy.

Although precision can well evaluate performances of different methods on a given network, it can’t evaluate the overall performances of different methods on different networks. Hence, relative precision is proposed to measure performances across different networks^27^. The random predictor is obtained by providing a ranking list that is ordered according to a random permutation of the links. So relative precision can be computed by


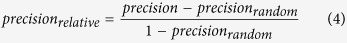


### Datasets and Baseline Algorithms

To test the performance of our proposed model, we consider the following 15 real world networks: C. elegans, the neural network of C. elegans^28^; Email, a communication network of human interaction^29^; Karate, the social networks of individuals of a karate club^30^; Word, an adjacency network of common adjectives and nouns in the novel David Copperfield by Charles Dickens^31^; Jazz, a network of jazz bands^32^; PB, the politicalblogs network of hyper-links between weblogs on politics^33^; USAir, the network of the USA airline^34^; Yeast, a network of Protein Protein Interaction on yeast^35^; NS, a network of coauthorships between scientists whose research centers on the properties of networks of one kind or another^31^; Power, the network representing the topology of the power grid of the US^36^; Router, a network of internet route^37^. Baydry, a food webs in the Florida Bay^38^; School, a friendship network in a high school^39^; SmaGri, a network of citation on network theory and experiment^34^; SW, a citation network on Physics^34^; The detail statistics of these networks are given in Table 1.

Next, we introduce some benchmark similarity methods as baselines for comparison, which are defined as following and each index is the similarity score of two nodes *x* and *y*.

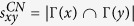
, where Γ(*x*) is the Neighbour nodes of *x*;
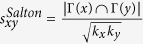
, where *k*_*x*_ is the degree of node *x*;
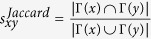
;
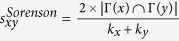
;
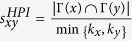
;
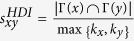
;
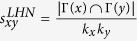
;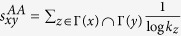;
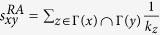
;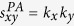;
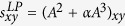
, where *α* is a parameter;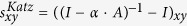, where *I* is the diagonal matrix and *α* is a parameter;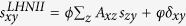, where *ϕ* and *φ* are parameters;
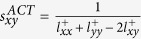
, which is denoted with Average Commute Time, where 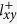 represents the elements of matrix *L*^+^ which is the pseudo inverse of the Laplacian matrix;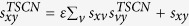, where *ε* is a parameter and *s*_*xy*_ has the same definition with 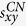.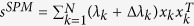, where *λ*_*k*_, *x*_*k*_ and Δ*λ*_*k*_ are the eigenvalue of the observed matrix, the corresponding orthogonal normalized eigenvector and the eigenvalue of a perturbation set respectively. Size of Δ*λ*_*k*_ is dependent on perturbation ratio *η*.
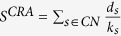
, where *k*_*s*_ is the degree of node x and *d*_*s*_ is the local-community degree of the common neighbour.

The detail definitions of the algorithms can be found in ref. 8 except *SPM* in ref. 23 and *CRA* in ref. 11. Note that five methods, including *LP, Katz, LHNII, TSCN* and *SPM*, are parameter-dependent and the others are parameter-free.

### Experiment results

We show the precision results of our proposed methods based on the perturbation-based framework and other baseline methods on the 15 real data sets in Table 2. The last row of Table 2 is the precision of a real random predictor which is obtained by providing a ranking list that is ordered according to a random permutation of the links. For every data set, the presented links are partitioned into training set (90%) and test set (10%). Ordinary *NMF* with Frobenius norm and ordinary *NMF* with KL divergence are denoted as *NMF*1 and *NMF*2, respectively. As shown in Table 2, *NMF* − *D*1 and *NMF* − *A*1 are better than *NMF*1, *NMF* − *D*2 and *NMF* − *A*2 are better than *NMF*2. As can be seen from Table 2, *NMF* − *A*2 has the best precision values on several real networks including C. elegans, USAir, Yeast, PB, Router, SmaGri, SW. *NMF* − *D*1 method has the best precision value on Karate network. Precisions of our proposed methods are very close to the highest ones, except for NS network and Power network. Overall, the proposed framework has competitive performance in real networks.

In addition, we also gave the respective precision-ranking position of each method in each network. Precision-ranking results of the proposed methods and other baseline methods are shown in Table 3. The last column of Table 3 is the mean ranking value of each method across all the networks and it is an indicator of average performance. In Table 3, different methods are presented in increasing order of mean precision-ranking. *NMF* − *A*2 has the best overall performance while *NMF* − *A*1, *NMF* − *D*1 and *NMF* − *D*2 have better average performance. Furthermore, in our proposed framework, *NMF* − *D*2 and *NMF* − *A*2 have lower precision-ranking values than *NMF* − *D*1 and *NMF* − *D*2, which suggests that performance of methods with KL divergence are better than those with Frobenius norm.

**Figure 2:**
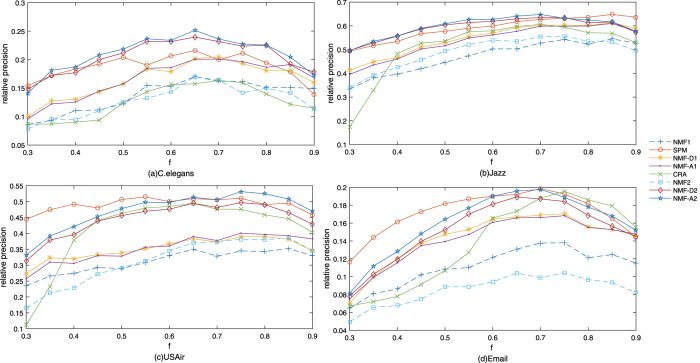
Comparison of relative precisions of methods under different fractions of training sets on four real networks. We compared relative precisions of eight methods under different fractions of training sets on the four networks and the precisions are returned with the average over 100 runs. The fraction of training sets *f* is varied from 0.3 to 0.9. The four networks are C. elegans, Jazz, USAir and Email. The link prediction methods are *NMF*1, *SPM, NMF* − *D*1, *NMF* − *A*1, *CRA, NMF*2, *NMF* − *D*2 and *NMF* − *A*2.

To accurately test the performance of our methods, the relative precision results of our proposed methods based on the perturbation-based framework and other baseline methods under different fractions of training sets in the different networks are shown in Fig. 2. As seen from Fig. 2, the methods with perturbation are better than those without perturbation as a whole. When the training set is very small (*f* = 0.3), the relative precision of *CRA* is lower than the other seven methods on Jazz network. The phenomenon that *CRA* lose performance for high level of network sparsification is a result of the fact that CRA is a local method based on the local communities that are cancelled by a heavy sparsification^11^. When we also plotted the LCP-corr values for different fractions of training set on the four networks(see Supplement Information, Fig. S1), we can see clearly that LCP-corr tends to increase with the higher fraction of training set. Bigger the LCP-corr is, better *CRA* method performs.

**Figure 3:**
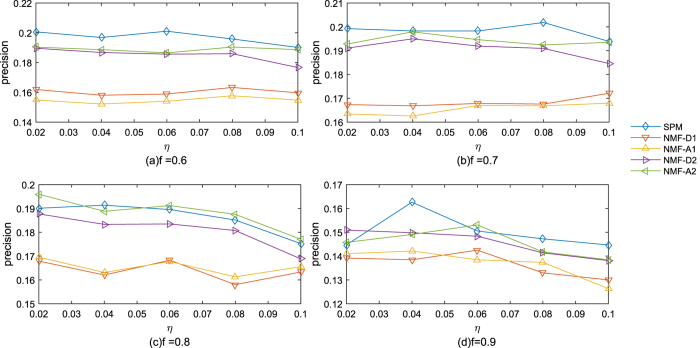
Comparison of precisions of five methods on different perturbation ratios on Email network. We compared precisions of all indices with perturbation on Email, which are *SPM, NMF* − *D*1, *NMF* − *A*1, *NMF* − *D*2 and *NMF* − *A*2. Different fractions of training set *f* are 0.6, 0.7, 0.8 and 0.9. The x-axis is perturbation ratio *η* varied from 0.02 to 0.1. The y-axis is the precision averaged over 100 independent runs.

We also give results under different ratios of perturbation set on Email, USAir, C. elegant, Jazz and Karate data sets. The result on Email data sets is shown in Fig. 3 and the results on USAir, C. elegant, Jazz and Karate data sets in Supplementary Information. As seen from Fig. 3, the precisions of *NMF* − *D*2 and *NMF* − *A*2 are significantly higher than the precisions of *NMF* − *D*1 and *NMF* − *A*1, which also shows that non-negative matrix factorization method with KL divergence is better than the non-negative matrix with Frobenius norm on the whole.

**Figure 4:**
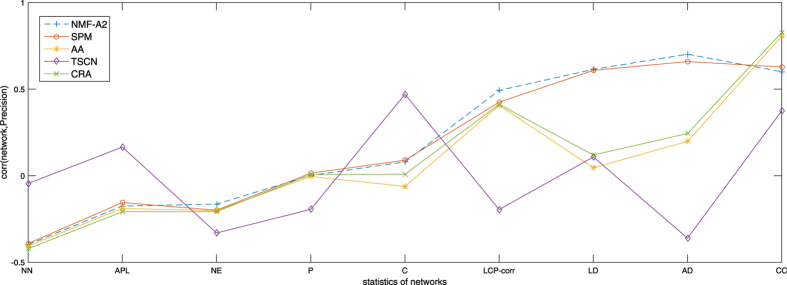
Correlation between precisions of four indices and statistics of networks where, NN, NE, LD, AD, APL, CC and P are the number of nodes, the number of edges, the link density, average degree, average shortest path length, clustering coefficient, and pearson assortative coefficient of the network, respectively. C is the average closeness for all the pair nodes of the network. LCP-corr is the correlation between LCP and CN indices presented in ref. 11.

As we know, the structure of a network has a strong influence on the result of link prediction. In order to find what kind of networks the different methods have well performances on, we calculate the correlation between precision and the statistics on different real data sets. The correlations between precisions of different methods and the statistic of networks are shown in Table 4 and the correlations between the precisions of four different methods and the statics of networks are shown in Fig. 4. The five methods are *NMF* − *A*2, *SPM, AA, CRA* and *TSCN*. For global methods, *NMF* − *A*2 method has the best overall performance and *SPM* method has the second best overall performance. For local methods, *CRA* method has the best overall performance and *AA* method has the second best overall performance. *TSCN* method is very unusual in aspect of correlation on statistics of networks. It can be seen from Fig. 4 that global methods with good precisions are very similar in aspect of correlation on statistics of networks, such as *SPM, NMF* − *A*2. They have positive correlations on average degree and clustering coefficient, which illustrates that their performances will be good when clustering coefficient and average degree of a network are large. They have negative correlations on number of nodes, which illustrates that their performances will be good when number of nodes of a network is small. Figure 4 also shows that local methods with good precisions are very similar, such as *CRA* and *AA*. But unlike global methods with good precisions, local methods with good precisions have little relation to average degree of a network.

## Discussion

In summary, real network is composed of predictable parts and unpredictable parts. Unpredictable parts includes noises and irregular links. In order to overcome prediction difficulties brought from these two kinds of unpredictable parts, we propose a perturbation framework based on non-negative matrix factorization, which can model the link behaviors from the latent feature information of networks. Based on this framework, we also proposed four methods which are called *NMF* − *D*1, *NMF* − *A*1, *NMF* − *D*2, *NMF* − *A*2, respectively.

We compared the proposed methods with other 19 baseline methods on 15 real data sets. These methods can be classified in different ways, such as glocal vs. local, parameter-dependent vs. parameter-free, and model-based vs. model-learning. Global methods require global topological information, however, local methods only make use of local topological information. *NMF*1, *NMF* − *D*1, *NMF* − *A*1, *NMF*2, *NMF* − *D*2, *NMF* − *A*2, *SPM, Katz, LHNII, ACT* and *TSCN* are all global methods. *Salton, Jaccard, Sorenson, HPI, HDI, LHN, CN, AA, RA, PA, LP, CRA* are all local methods. The global methods perform better, but the complexity is higher. The local methods are suitable for large-scale networks due to the trade off between complexity and performance. Among the global methods, *NMF* − *A*2 has the best precision values on several data sets including C. elegant, Email, USAir, Yeast, PB, Router, SmaGri and SW. *NMF* − *A*2 also has the second best precision values on Baydry network and School network. *NMF* − *D*1 has the best precision on Karate network and its precision is very close to the highest one on Jazz network. As can be seen from Table 3, *NMF* − *A*2 is the best global method and *SPM* is the second best global method. Among the local methods, *CRA* has the best precision values on several networks including C. elegant, Email, Karate, Jazz, Yeast, PB, Router, School, SmaGri and SW (Table 2) and it also has the best mean ranking value (Table 3). Hence, *CRA* is the best local method.

Parameter-free methods are those methods without parameter to tune and parameter-dependent methods are those methods with several parameters to tune. In the 23 method, *NMF* − *D*1, *NMF* − *A*1, *NMF* − *D*2, *NMF* − *A*2, *SPM, Katz, LHNII, LP, TSCN* are parameter-dependent methods and the other methods are parameter-free methods. *NMF* − *A*2 is the best parameter-dependent method and *CRA* is the best parameter-free method because they have the lowest mean precision-ranking values for each of these classes. As a whole, parameter-dependent methods have better average performances than parameter-free methods. However, the inevitable problem of parameter-dependent methods is that tuning of parameters is still an obstacle for practical applications because in many cases it is not clear how to tune the parameters.

In addition, methods also can be divided into two categories: model-based and model-learning. Model-based methods are based on an explicit deterministic model that simulates physical mechanism behind the network organization. Model-learning methods are based on implicit model-learning: providing at every step a different solution that can converge to hidden the network evolution by many times of iterations^12^. *NMF*1, *NMF*2, *NMF* − *D*1, *NMF* − *A*1, *NMF* − *D*2, *NMF* − *A*2, *SPM* are model-learning methods and the other methods are model-based methods. Among model-learning methods, *NMF* − *A*2 is the best model-learning method and *SPM* is the second best model-learning method. Among model-based methods, *CRA* is the best model-based method and *AA* is the second best model-based method. Most of model-learning methods usually are parameter-dependent. Although model-learning methods perform better than model-based methods, model-learning methods have higher computational time. In general, experimental results show that the proposed methods have better and stable performance compared with baseline methods on 15 data sets.

We also find that those methods with perturbation perform better than ordinary methods on almost of all networks. Furthermore, NMF with KL divergence is more suitable for link prediction than NMF with Frobenius norm. In short, experiment results demonstrate that our framework is effective.

In the future, the proposed framework could be further improved. For example, NMF needs iterative calculation, which result in high complexity. Parallelization and sampling methods can be adopted to reduce the computational complexity. NMF may obtain the local optimal solution, so how to get the global optimal solution is also a challenging issue. Although to some extent, the perturbation framework can alleviate the problem from noises and irregular links, it remains an open problem to find out the unpredictable parts objectively.

## Methods

### Method and algorithm of perturbation-based framework

#### Determination of the number of latent features by Colibri

There are many methods to determine the number of latent features, such as Bayesian information Criterion (BIC) and cross validation, which need to calculate each possible value of the number of latent features and are not suitable in real networks. Another method called Bayesian non-negative matrix factorization^40^ which is based on the automatic relevance determination. However, all these methods are computational complexity, so we determine the number of latent features by Colibri^24^ used for low-rank approximations of the adjacency matrix of a graph. The main idea is to eliminate linearly dependent columns while iterating over sampled columns for low rank approximation.

#### Calculation of common basis matrix *W* and coefficients matrix *H*

To optimize the cost functions *O*_1_ in (1) and *O*_2_ in (2), we utilize the simple multiplicative update method^41^ for NMF. The update rule for *O*_1_ is as follows


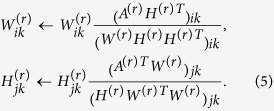


The algorithm minimizing the cost function *O*_2_ is as follows


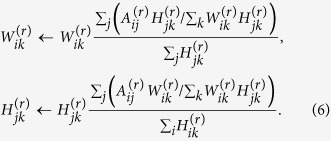


It is easy to prove that the above two update rules will find local minima of the cost functions *O*_1_ and *O*_2_^41^.

### The algorithm of the proposed framework


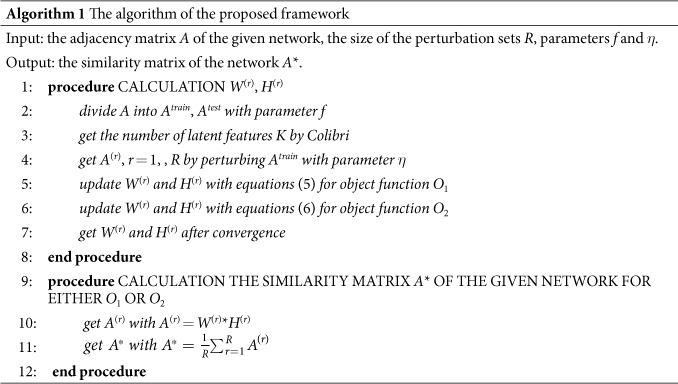


We can obtain the similarity matrix A* by the above optimal procedures and the pseudocode is presented in algorithm 1.

## Additional Information

**How to cite this article**: Wang, W. *et al*. A perturbation-based framework for link prediction via non-negative matrix factorization. *Sci. Rep.* **6**, 38938; doi: 10.1038/srep38938 (2016).

**Publisher's note:** Springer Nature remains neutral with regard to jurisdictional claims in published maps and institutional affiliations.

## Supplementary Material

Supplementary Information

Supplementary Dataset 1

## Figures and Tables

**Table 1:** Statistics of the networks studied in this paper.

Network	NN	NE	LD	AD	APL	C	CC	P	LCP-corr
C. elegans	297	2148	0.0489	14.4646	2.4553	0.0014	0.2924	−0.1632	0.9056
Email	1133	5451	0.0085	9.6222	3.6060	0.0002	0.1663	0.0782	0.8538
Karate	34	78	0.1390	4.5882	2.4082	0.0129	0.5706	−0.4756	0.7562
Word	112	425	0.0684	7.5893	2.5356	0.0036	0.1728	−0.1293	0.8528
Jazz	198	2742	0.1406	27.6970	2.2350	0.0023	0.6175	0.0202	0.9485
USAir	332	2126	0.0387	12.8072	2.7381	0.0011	0.6252	−0.2079	0.9799
Yeast	2361	6646	0.0024	5.6298	5.0960	0.0001	0.3057	0.4539	0.9686
PB	1222	16714	0.0224	27.3552	2.7375	0.0003	0.3203	−0.2213	0.9286
NS	379	914	0.0128	4.8232	6.0419	0.0004	0.7412	−0.0817	0.9224
Power	4941	6594	0.0005	2.6691	18.9892	0.0000	0.1032	0.0035	0.8456
Router	5022	6258	0.0005	2.4922	6.4488	0.0000	0.0303	−0.1384	0.8067
Baydry	128	2106	0.2591	32.9063	1.7724	0.0045	0.3346	−0.1044	0.9112
School	69	220	0.09378	6.3768	2.965	0.005	0.4606	0.0141	0.9005
SmaGri	1024	4916	0.0094	9.6016	2.9814	0.0003	0.3071	−0.1925	0.9463
SW	233	994	0.03678	8.5322	2.3711	0.0018	0.5564	−0.3027	0.9436

Where, NN, NE, LD, AD, APL, CC and P are the number of nodes, the number of edges, the link density, average degree, average shortest path length, clustering coefficient, and pearson assortative coefficient of the network, respectively. C is the average closeness for all the pair nodes of the network, LCP-corr is the correlation between LCP and CN indices presented in ref. 11.

**Table 2:** Precision values of different methods on 15 networks.

Precision	C. elegant	Email	Karate	Word	Jazz	USAir	Yeast	PB	NS	Router	Power	Baydry	School	SmaGri	SW
NMF1*	*0*.*142*	*0*.*111*	*0*.*201*	*0*.*042*	*0*.*548*	*0*.*320*	*0*.*139*	*0*.*143*	*0*.*265*	*0*.*025*	*0*.*022*	*0*.*477*	*0*.*195*	*0*.*053*	*0*.*123*
*NMF*-D1*(*2*)	*0*.*171*	*0*.*143*	***0***.***234***	*0*.*042*	*0*.*615*	*0*.*362*	*0*.*165*	*0*.*170*	*0*.*298*	*0*.*016*	*0*.*023*	*0*.*539*	*0*.*216*	*0*.*068*	*0*.*153*
*NMF*-A1*(*2*)	*0*.*175*	*0*.*143*	*0*.*214*	*0*.*042*	*0*.*605*	*0*.*386*	*0*.*168*	*0*.*170*	*0*.*311*	*0*.*021*	*0*.*026*	*0*.*541*	*0*.*218*	*0*.*068*	*0*.*153*
NMF2*	*0*.*122*	*0*.*075*	*0*.*183*	*0*.*055*	*0*.*512*	*0*.*350*	*0*.*079*	*0*.*158*	*0*.*202*	*0*.*103*	*0*.*018*	*0*.*430*	*0*.*168*	*0*.*049*	*0*.*165*
*NMF*-D2*(*2*)	*0*.*185*	*0*.*144*	*0*.*201*	*0*.*079*	*0*.*593*	*0*.*445*	*0*.*169*	*0*.*234*	*0*.*303*	*0*.*067*	*0*.*029*	*0*.*520*	*0*.*182*	*0*.*118*	*0*.*246*
*NMF*-A2*(*2*)	***0***.***191***	***0***.***149***	*0*.*201*	*0*.*080*	*0*.*600*	***0***.***470***	***0***.***186***	***0***.***248***	*0*.*310*	***0***.***235***	*0*.*036*	*0*.*544*	*0*.*218*	***0***.***130***	***0***.***276***
SPM*(*2*)	*0*.*171*	*0*.*144*	*0*.*210*	***0***.***101***	***0***.***650***	*0*.*449*	*0*.*160*	*0*.*238*	***0***.***420***	*0*.*224*	*0*.*057*	***0***.***552***	***0***.***227***	*0*.*118*	*0*.*211*
*Katz*(*1*)	*0*.*102*	*0*.*131*	*0*.*169*	*0*.*072*	*0*.*449*	*0*.*365*	*0*.*108*	*0*.*175*	*0*.*299*	*0*.*060*	***0***.***058***	*0*.*085*	*0*.*142*	*0*.*099*	*0*.*151*
*LHNII*(*2*)	*0*.*000*	*0*.*000*	*0*.*000*	*0*.*001*	*0*.*047*	*0*.*003*	*0*.*000*	*0*.*000*	*0*.*008*	*0*.*000*	*0*.*010*	*0*.*005*	*0*.*062*	*0*.*000*	*0*.*001*
*ACT*	*0*.*053*	*0*.*024*	*0*.*128*	*0*.*087*	*0*.*169*	*0*.*332*	*0*.*000*	*0*.*077*	*0*.*193*	*0*.*160*	*0*.*034*	*0*.*118*	*0*.*142*	*0*.*035*	*0*.*101*
*TSCN*(*1*)	*0*.*018*	*0*.*014*	*0*.*145*	*0*.*002*	*0*.*024*	*0*.*133*	*0*.*032*	*0*.*027*	*0*.*087*	*0*.*096*	*0*.*056*	*0*.*036*	*0*.*197*	*0*.*028*	*0*.*039*
Salton	0.024	0.050	0.001	0.001	0.535	0.046	0.000	0.013	0.253	0.000	0.015	0.011	0.175	0.000	0.001
Jaccard	0.028	0.071	0.001	0.002	0.521	0.064	0.000	0.017	0.252	0.000	0.007	0.010	0.180	0.000	0.001
Sorenson	0.028	0.065	0.001	0.002	0.521	0.064	0.000	0.017	0.252	0.000	0.009	0.010	0.180	0.000	0.001
HPI	0.015	0.007	0.091	0.005	0.255	0.016	0.012	0.003	0.146	0.000	0.005	0.055	0.105	0.002	0.000
HDI	0.029	0.069	0.004	0.004	0.465	0.083	0.000	0.025	0.264	0.000	0.007	0.009	0.173	0.000	0.001
LHN	0.000	0.003	0.004	0.000	0.093	0.004	0.000	0.000	0.084	0.000	0.010	0.014	0.082	0.000	0.001
CN	0.095	0.139	0.164	0.064	0.509	0.372	0.104	0.174	0.379	0.057	**0**.**051**	0.065	0.162	0.090	0.112
AA	0.112	0.151	0.163	0.067	0.524	0.396	0.104	0.172	0.563	0.038	0.030	0.063	0.148	0.103	0.131
RA	0.112	0.138	0.165	0.056	0.545	**0**.**473**	0.083	0.151	**0**.**586**	0.020	0.030	0.065	0.187	0.102	0.139
PA	0.060	0.014	0.096	**0**.**089**	0.130	0.318	0.012	0.069	0.012	0.025	0.001	**0**.**167**	0.025	0.051	0.099
LP(1)	0.100	0.131	0.169	0.072	0.495	0.370	0.107	0.175	0.299	0.059	0.054	0.071	0.113	0.095	0.128
CRA	**0**.**116**	**0**.**157**	**0**.**199**	0.038	**0**.**557**	0.391	**0**.**123**	**0**.**177**	0.481	**0**.**062**	0.033	0.085	**0**.**210**	**0**.**118**	**0**.**147**
Random	0.005	8.5e-4	0.016	0.007	0.016	0.004	2.3e-4	0.002	0.001	5e-5	5.4e-5	0.034	0.010	0.001	0.004

We compared our methods with other methods on the 15 network data sets and the precisions are returned with the average over 100 runs. The last row is the precision value of a real random predictor which is obtained by providing a ranking list that is ordered according to a random permutation of the links. For every data set, the presented links are partitioned into training set (90%) and test set (10%). The local methods are in standard character while the global methods are in italic. Number in bracket closed to a method denotes the number of tuning parameters. The best result achieved by global methods and the best result achieved by local methods on each network are boldface. Methods with an asterisk like * denote methods based on inference and methods without an asterisk denote methods based on a paradigm (in the sense that are model-based). We tune the parameters to optimize the performance of baseline methods for comparison. In our experiments, we set *α* = 0.0001 for *LP*, parameter *α* = 0.01 for Katz, *ϕ* = 0.99 and *φ* = 1 for *LHNII, η* = 0.1 for *SPM, NMF* − *D*1, *NMF* − *A*1, *NMF* − *D*2 and *NMF* − *D*2.

**Table 3:** Precision-ranking of the different network.

	C. elegant	Email	Karate	Word	Jazz	USAir	Yeast	PB	NS	Router	Power	Baydry	School	SmaGri	SW	mean
***NMF***-A2*(***2***)	*1*	*3*	*4*	*4*	*4*	*2*	*1*	*1*	*7*	*1*	*6*	*2*	*2*	*1*	*1*	***2***.***67***
SPM*(*2*)	*5*	*5*	*3*	*1*	*1*	*3*	*5*	*2*	*4*	*2*	*2*	*1*	*1*	*3*	*3*	*2*.*73*
*NMF*-D2*(*2*)	*2*	*4*	*5*	*5*	*5*	*4*	*2*	*3*	*8*	*6*	*11*	*5*	*9*	*2*	*2*	*4*.*87*
**CRA**	8	1	7	15	6	6	7	4	3	7	8	11	5	4	8	**6**.**67**
*NMF*-A1*(*2*)	*3*	*7*	*2*	*14*	*3*	*7*	*3*	*10*	*6*	*14*	*12*	*3*	*3*	*11*	*6*	*6*.*93*
*NMF*-D1*(*2*)	*4*	*6*	*1*	*13*	*2*	*11*	*4*	*9*	*11*	*16*	*13*	*4*	*4*	*10*	*5*	*7*.*53*
AA	9	2	13	8	10	5	11	8	2	11	9	15	16	5	10	8.93
RA	10	9	11	10	8	1	12	12	1	15	10	14	8	6	9	9.07
*Katz*(*1*)	*11*	*11*	*10*	*7*	*17*	*10*	*8*	*6*	*10*	*8*	*1*	*10*	*17*	*7*	*7*	*9*.*33*
LP(1)	12	10	9	6	15	9	9	5	9	9	4	12	19	8	11	9.80
NMF1*	*6*	*12*	*6*	*12*	*7*	*14*	*6*	*13*	*12*	*12*	*14*	*6*	*7*	*12*	*12*	*10*.*07*
CN	13	8	12	9	14	8	10	7	5	10	5	13	15	9	13	10.07
NMF2*	*7*	*13*	*8*	*11*	*13*	*12*	*13*	*11*	*17*	*4*	*15*	*7*	*14*	*14*	*4*	*10*.*87*
*ACT*	*15*	*18*	*15*	*3*	*19*	*13*	*23*	*14*	*18*	*3*	*7*	*9*	*18*	*15*	*14*	*13*.*60*
*TSCN*(*1*)	*20*	*20*	*14*	*20*	*23*	*16*	*14*	*16*	*20*	*5*	*3*	*17*	*6*	*16*	*16*	*15*.*07*
PA	14	19	16	2	20	15	16	15	22	13	23	8	23	13	15	15.60
Jaccard	17	14	21	18	11	18	18	18	15	18	20	20	10	19	18	17.00
Salton	19	17	20	21	9	20	17	20	14	17	16	19	12	18	17	17.07
HDI	16	15	18	17	16	17	20	17	13	21	21	22	13	21	20	17.80
Sorenson	18	16	22	19	12	19	19	19	16	19	19	21	11	20	19	17.93
HPI	21	21	17	16	18	21	15	21	19	20	22	16	20	17	23	19.13
LHN	22	22	19	23	21	22	21	22	21	22	17	18	21	22	21	20.93
*LHNII*(*2*)	*23*	*23*	*23*	*22*	*22*	*23*	*22*	*23*	*23*	*23*	*18*	*23*	*22*	*23*	*22*	*22*.*33*

The last column is the mean precision-ranking across all the networks. Different methods are presented in increasing order of mean precision-ranking. The local methods are in standard character while the global methods are in italic. The name of the best local method and the name of the best global method in the first column are boldface. Corresponding, the mean precision-ranking value of the best local method and the mean precision-ranking value of the best global method in the last column also are boldface. Number in bracket closed to an method denotes the number of tuning parameters. The best result achieved by global methods and the best result achieved by local methods on each network are boldface. Methods with an asterisk like * denote methods based on inference and methods without an asterisk denote methods based on a paradigm (in the sense that are model-based).

**Table 4:** Correlation between precisions of different methods and the statistic of networks.

	NN	NE	LD	AD	APL	C	CC	P	LCP-corr
NMF1	−0.51	−0.26	0.73	0.70	−0.38	0.24	0.63	0.03	0.37
NMF-D1	−0.53	−0.26	0.73	0.71	−0.41	0.24	0.64	0.02	0.38
NMF-A1	−0.52	−0.26	0.71	0.71	−0.40	0.20	0.64	0.03	0.41
NMF2	−0.46	−0.23	0.71	0.71	−0.28	0.22	0.61	−0.08	0.38
NMF-D2	−0.53	−0.20	0.65	0.74	−0.40	0.13	0.66	−0.04	0.50
NMF-A2	−0.40	−0.16	0.61	0.70	−0.18	0.08	0.60	0.00	0.49
SPM	−0.39	−0.20	0.61	0.66	−0.16	0.09	0.63	0.01	0.42
Salton	−0.27	−0.21	0.24	0.30	−0.13	−0.02	0.53	0.20	0.27
Jaccard	−0.29	−0.22	0.23	0.30	−0.14	−0.03	0.54	0.20	0.28
Sorenson	−0.29	−0.22	0.23	0.30	−0.14	−0.03	0.54	0.20	0.28
HPI	−0.37	−0.35	0.46	0.29	−0.19	0.30	0.63	0.06	0.09
HDI	−0.30	−0.22	0.20	0.28	−0.14	−0.04	0.58	0.19	0.29
LHN	−0.28	−0.33	0.25	0.13	−0.10	0.07	0.56	0.19	0.19
CN	−0.39	−0.16	0.11	0.28	−0.21	−0.01	0.77	0.00	0.40
AA	−0.41	−0.20	0.05	0.20	−0.19	−0.06	0.81	−0.01	0.41
RA	−0.42	−0.25	0.06	0.18	−0.21	−0.04	0.82	−0.04	0.42
PA	−0.44	−0.23	0.43	0.45	−0.35	0.19	0.44	−0.35	0.36
LP	−0.38	−0.13	0.14	0.34	−0.24	0.00	0.74	−0.03	0.42
Katz	−0.42	−0.15	0.14	0.32	−0.27	0.01	0.79	−0.06	0.44
LHNII	−0.21	−0.27	0.31	0.14	−0.15	0.16	0.29	0.21	0.13
ACT	−0.26	−0.33	0.21	0.10	0.09	0.15	0.61	−0.28	0.23
TSCN	−0.05	−0.33	0.11	−0.36	0.17	0.47	0.37	−0.19	−0.20
CRA	−0.42	−0.21	0.12	0.24	−0.21	0.01	0.83	0.01	0.41

NN, NE, LD, AD, APL, CC and P are the number of nodes, the number of edges, the link density, average degree, average shortest path length, clustering coefficient, and pearson assortative coefficient of the network, respectively, C is the average closeness for all the pair nodes of the network. LCP-corr is the correlation between LCP and CN presented in ref. 11.
