# Supplementary material for: A perturbation-based framework for link prediction via non-negative matrix factorization
Source: Sci Rep. 2016 Dec 15;6:38938. doi: 10.1038/srep38938 (PMC5156920; doi:10.1038/srep38938)
Supplement: Supplementary Information [file srep38938-s1.pdf]

A perturbation-based framework for link prediction via non-negative matrix factorization

Wenjun Wang, Fei Cai, Pengfei Jiao and Lin Pan

Supplementary Information

1 Supplement Figures

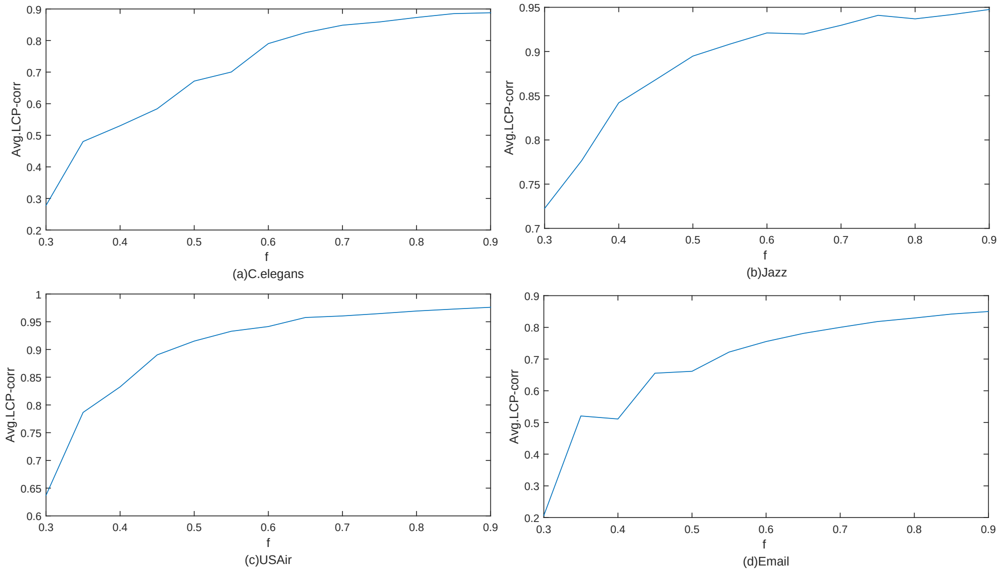

Figure S1. Average LCP-corr under different fractions of training sets on four real networks. The four real networks are C.elegans, Jazz, USAir and Email. The x-axis is the fraction of training set varied from 0.3 to 0.9. The y-axis is the LCP-corr averaged over 100 independent runs.

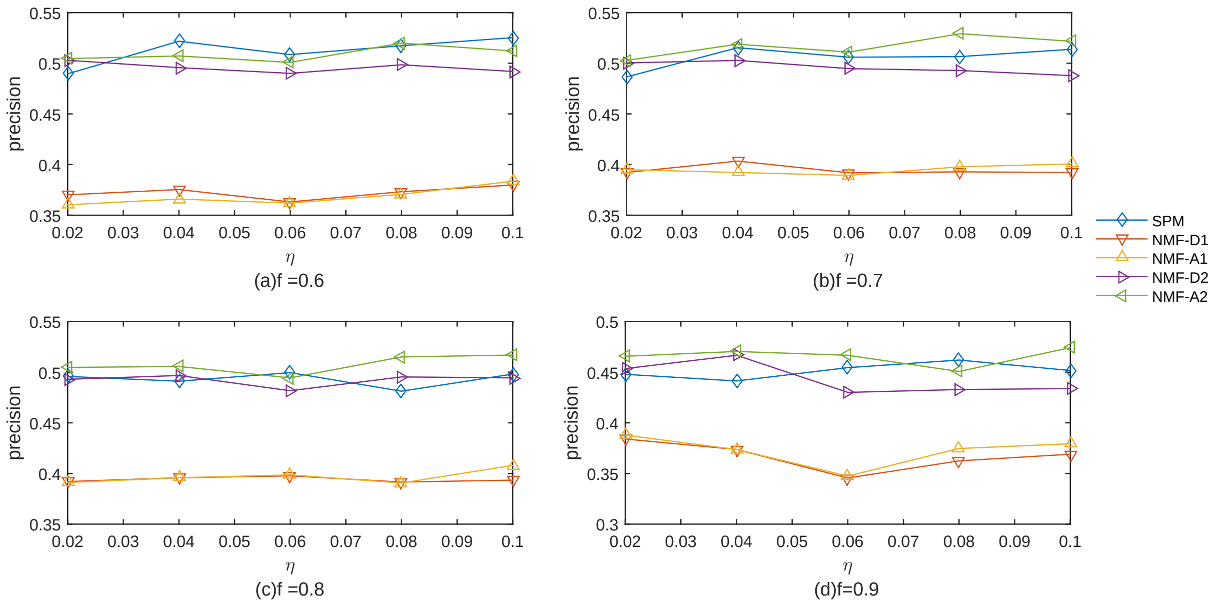

Figure S2. Comparison of precisions of five methods under different perturbation ratios and the different fractions of training sets on USAir network. We compared precisions of all indices with perturbation on Email, which are SPM, $NMF-D1$, $NMF-A1$, $NMF-D2$ and $NMF-A2$. different fractions of training set f are 0.6, 0.7,0.8 and 0.9. The x-axis is perturbation ratio η varied from 0.02 to 0.1. The y-axis is the precision averaged over 100 independent runs.

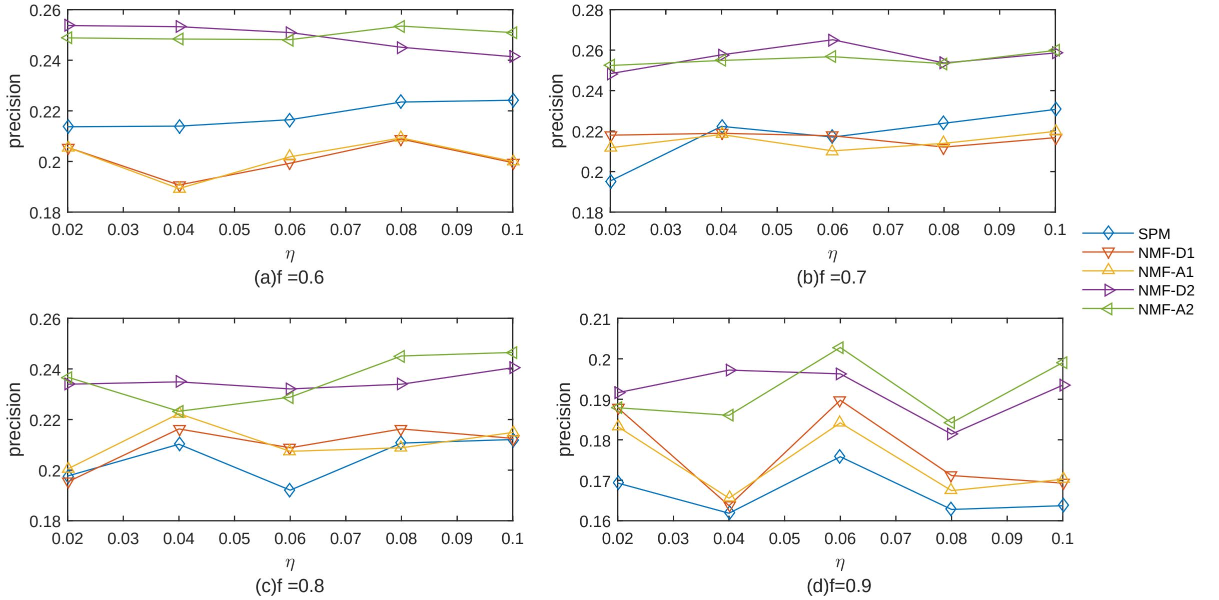

Figure S3. Comparison of precisions of five methods under different perturbation ratios and the different fractions of training sets on C.elegant network. We compared precisions of all indices with perturbation on Email, which are SPM, $NMF-D1$, $NMF-A1$, $NMF-D2$ and $NMF-A2$. The different fractions of training set f are 0.6, 0.7,0.8 and 0.9. The x-axis is perturbation ratio η varied from 0.02 to 0.1. The y-axis is the precision averaged over 100 independent runs.

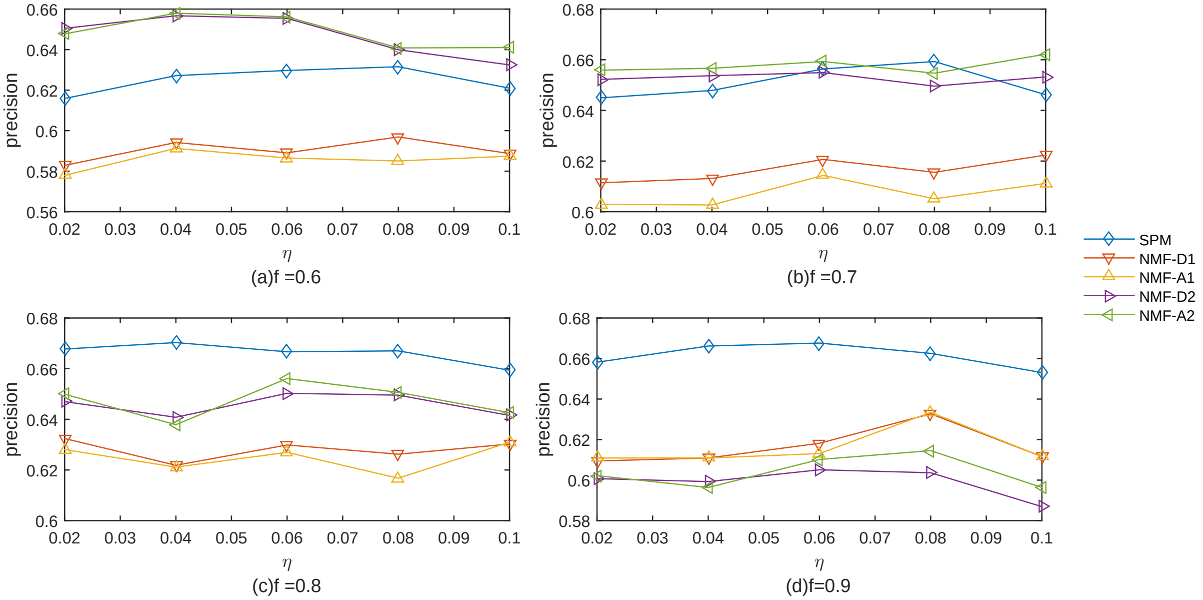

Figure S4. Comparison of precisions of five methods under different perturbation ratios and the different fractions of training sets on Jazz network. We compared precisions of all indices with perturbation on Email, which are SPM, $NMF-D1$, $NMF-A1$, $NMF-D2$ and $NMF-A2$. The different fractions of training set f are 0.6, 0.7,0.8 and 0.9. The x-axis is perturbation ratio η varied from 0.02 to 0.1. The y-axis is the precision averaged over 100 independent runs.

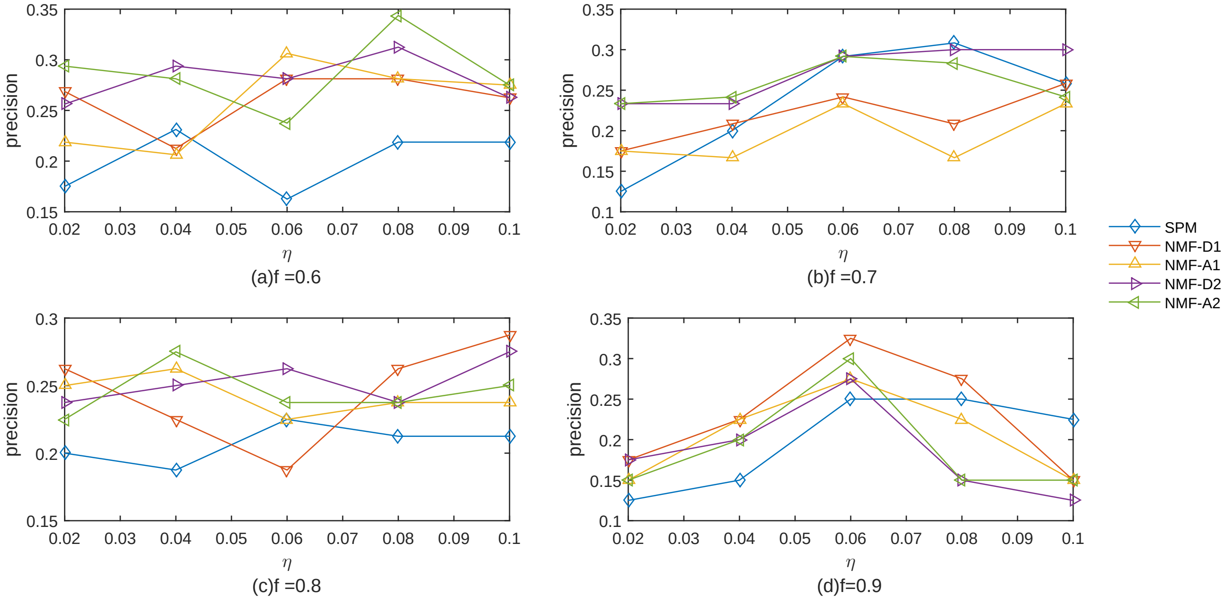

Figure S5. Comparison of precisions of five methods under different perturbation ratios and the different fractions of training sets on Karate network. We compared precisions of all indices with perturbation on Email, which are SPM, $NMF-D1$, $NMF-A1$, $NMF-D2$ and $NMF-A2$. The different fractions of training set f are 0.6, 0.7,0.8 and 0.9. The x-axis is perturbation ratio η varied from 0.02 to 0.1. The y-axis is the precision averaged over 100 independent runs.

2 Link for Code Download

We have provided the MATLAB code to compute the our proposed four methods, which are $NMF-D1$, $NMF-A1$, $NMF-D2$ and $NMF-A2$, respectively. MATLAB code is submitted as supplementary data file and also is provided at the following link: http://www.pedsurgcn.com/data/PerturbProgram.zip.
